# Editorial: Viral Interactions with the Nucleus

**DOI:** 10.3389/fmicb.2017.00951

**Published:** 2017-05-26

**Authors:** Erin J. Walker, Reena Ghildyal

**Affiliations:** Health Research Institute, University of CanberraCanberra, ACT, Australia

**Keywords:** DNA viruses, RNA viruses, nuclear pore complex, viral nuclear interactions, nuclear transport

The eukaryotic genetic material is sequestered within the nucleus bound by the nuclear envelope (NE), separating the genetic material, and its functions from the surrounding cytoplasm. Regulated transport of macromolecules through the nuclear pore complex (NPC), the only means of transport across the nuclear envelope, is essential for normal cell function and effective antiviral responses. Many viruses disrupt or exploit the host cell nucleocytoplasmic trafficking pathways in order to access nuclear functions.

This research topic has assembled reviews and original research articles demonstrating the diversity and importance of viral interactions with the nucleus. The viruses range from DNA, RNA viruses, to enveloped, non-enveloped viruses, and include retroviruses, demonstrating that exploitation of the host nuclear process is a common theme across diverse virus families. All articles in this topic address viruses of veterinary and/or medical importance.

The importance of nuclear entry is addressed in a review focusing on enveloped and non-enveloped DNA viruses (Fay and Panté), which deliver their genome into the nucleus for replication. Intact capsids of hepatitis B virus (HBV) are transported into the nucleus via translocation through the NPC. In contrast, herpes simplex virus 1 (HSV1) docks on the cytoplasmic side of the NPC, enabling release of the viral genome into the nucleus. Non-enveloped DNA viruses deliver their genomes into the nucleus in diverse ways, including docking at the NPC (adenovirus), disruption of the NE (parvovirus), and accessing the nucleus during breakdown of the NE in mitosis (human papillomavirus).

Most RNA viruses do not strictly require nuclear entry of their genome to replicate, but may require RNA-binding proteins found in the nucleus for replication. Entry of RNA virus proteins such as proteases into the nucleus also enables disruption of host-cell transcription and innate anti-viral responses, as discussed in the review on picornaviral nuclear interactions (Flather and Semler). The picornaviral theme is developed further by Walker et al. who describe the nuclear effect of two serotypes of human rhinovirus (HRV), a member of the picornavirus family (Walker et al.). While both serotypes lead to changes in NPC proteins and redistribution of nuclear proteins, this study demonstrates the variation that occurs between family members.

Influenza virus, HRV, and respiratory syncytial virus (RSV) are the main viral causative agents of respiratory infectious disease and all three exploit the nucleocytoplasmic trafficking mechanisms, albeit in different ways (Caly et al.). Influenza virus replication occurs at viral ribonucleocapsid complexes in the nucleus. HRV disrupts nucleocytoplasmic trafficking by directed cleavage of NPC proteins by viral proteases, disrupting nuclear transport, and providing viral access to nuclear proteins required to support HRV replication. RSV uses nuclear transport pathways to move its matrix protein into and out of the nucleus at specific times in infection to facilitate virus replication and assembly.

Nucleocapsid proteins of some enveloped RNA viruses localize to the nucleus of infected cells; this is intriguing as these proteins are essential for virus replication and assembly in the cytoplasm. A review of the nucleocytoplasmic transport of these proteins and their proposed nuclear functions is provided by the Ghildyal group, detailing the nuclear transport signals present in members of the *Flaviviridae, Coronaviridae, Arteriviridae*, and *Paramyxoviridae* families (Wulan et al.).

As well as the nucleocapsid (or core protein) of the hepatitis C virus (HCV), a number of non-structural proteins carry nuclear localization signals and are found in the nucleus during infection, as reviewed by Bonamassa et al. The functional roles of non-structural protein NS5A are varied, including biogenesis of viral replication factories, viral replication and assembly of viral particles (Bonamassa et al.), potentially acting as a switch between viral replication, and particle assembly. As described for many viruses, the HCV NS5A protein affects host interferon responses by interfering with transcriptional regulation of relevant genes.

The host cell nucleus is essential to retrovirus replication as the reverse transcribed DNA is integrated into the host genome where it is transcribed by the host machinery. Rice et al. show that the Gag protein may co-opt cellular splicing signals to ensure encapsidation of the unspliced genomic viral RNA (gRNA). Previous work by the group had shown nuclear trafficking of Gag is required for efficient encapsidation of gRNA. The current article (Rice et al.) takes their work and sheds light on the nuclear functions of Gag. Their work raises the possibility that Gag localizes to the nucleus and associates with transcription factories to gain access to nascent unspliced viral RNA in order to capture it for encapsidation.

Herpes viruses, enteroviruses, and flaviviruses comprise majority of the known neurotrophic viruses. Interestingly, despite their very different genomes and diverse replication cycles, many have very similar pathogenic mechanisms. In their review, Hill et al. discuss the induction of a key micro-RNA (miRNA) by several neurotrophic viruses. miRNA-146a is induced early in infection via an NF-κB dependent pathway. As NF-κB is a mediator of proinflammatory and antiviral pathways, the question arises as to whether the induction of miRNA-146a is a protective host response or supports the virus infection; this is not yet known. It is however clear that miRNA-146a is involved in the observed pathogenic responses to infection and may have a role in the observed modulation of inflammation in antiviral treatment (Hill et al.).

The article by Tu and Rao addresses the important topic of immunosenescence in T cells. We have known for some time that the immune system undergoes cumulative age-associated changes, which produce a progressive deterioration in the ability to respond to infections and to develop immunity after vaccination. Modification of nuclear functions via epigenetic changes induced by latent cytomegalovirus infection is implicated in pathological changes in T cells that accelerate age-associated immunosenescence (Tu and Rao). Vaccine efficiency in the elderly may be enhanced by targeting the epigenetic changes induced by cytomegalovirus infection.

Together these papers demonstrate the importance of interactions with the nucleus across a range of different viruses of medical importance; whether by hijacking nuclear transport pathways, disrupting host nuclear transport to limit immune responses, or access specific nuclear proteins.

## Author contributions

All authors listed, have made substantial, direct and intellectual contribution to the work, and approved it for publication.

### Conflict of interest statement

The authors declare that the research was conducted in the absence of any commercial or financial relationships that could be construed as a potential conflict of interest.

